# Delayed Effect of Acupuncture Treatment in OA of the Knee: A Blinded, Randomized, Controlled Trial

**DOI:** 10.1093/ecam/nen080

**Published:** 2010-10-20

**Authors:** Ehud Miller, Yair Maimon, Yishai Rosenblatt, Anat Mendler, Avi Hasner, Adi Barad, Hagay Amir, Shmuel Dekel, Shahar Lev-Ari

**Affiliations:** ^1^Unit of Complementary Medicine, Tel Aviv Sourasky Medical Center, Sackler Faculty of Medicine, Tel Aviv University, Tel Aviv, Israel; ^2^Department of Orthopaedic Surgery “B”, Tel Aviv Sourasky Medical Center, Sackler Faculty of Medicine, Tel Aviv University, Tel Aviv, Israel; ^3^Unit of Quality Control, Tel Aviv Sourasky Medical Center, Sackler Faculty of Medicine, Tel Aviv University, Tel Aviv, Israel

## Abstract

To assess the efficacy in providing improved function and pain relief by administering 8 weeks of acupuncture as adjunctive therapy to standard care in elderly patients with OA of the knee. This randomized, controlled, blinded trial was conducted on 55 patients with OA of the knee. Forty-one patients completed the study (26 females, 15 males, mean age ± SD 71.7 ± 8.6 years). Patients were randomly divided into an intervention group that received biweekly acupuncture treatment (*n* = 28) and a control group that received sham acupuncture (*n* = 27), both in addition to standard therapy, for example, NSAIDS, cyclooxygenase-2 inhibitors, acetaminophen, intra-articular hyaluronic acid and steroid injections. Primary outcomes measures were changes in the Knee Society Score (KSS) knee score and in KSS function and pain ratings at therapy onset, at 8 weeks (closure of study) and at 12 weeks (1 month after last treatment). Secondary outcomes were patient satisfaction and validity of sham acupuncture. There was significant improvement in all three scores in both groups after 8 and 12 weeks compared with baseline (*P* < .05). Significant differences between the intervention and control groups in the KSS knee score (*P* = .036) was apparent only after 12 weeks. Patient satisfaction was higher in the intervention group. Adjunctive acupuncture treatment seems to provide added improvement to standard care in elderly patients with OA of the knee. Future research should determine the optimal duration of acupuncture treatment in the context of OA.

## 1. Introduction

OA is a systemic and chronic inflammatory disorder for which there is no curative treatment [1, 2]. It is characterized by degradation of articular cartilage and is associated with loss of mobility and the consequent loss of functional independence [3]. OA is the leading cause of disability among the elderly, and it has been estimated that 68% of Americans older than 55 years of age are affected [4].

Current pharmacologic treatments for OA include NSAIDs, cyclooxygenase-2 (COX-2) inhibitors, acetaminophen, intra-articular hyaluronic acid and steroid injections. These drugs, however, did not generally prove to have substantial disease-modifying efficacy, and were often shown to have toxic effects after chronic administration [5, 6].

Originating in China more than 2000 years ago, acupuncture is one of the oldest, most commonly used medical procedures in the world. The term acupuncture describes a family of procedures involving the stimulation of anatomical points on the body by a variety of techniques in order to exert a therapeutic effect. The acupuncture technique that has been most studied scientifically in osteoarthritis involves penetrating the skin with thin, solid, metallic needles that are manipulated by the practitioner's hands (manual acupuncture) or by electrical stimulation (electro-acupuncture). According to the large scale 2002 National Health Interview Survey, an estimated 8.2 million US adults had ever used acupuncture, and an estimated 2.1 million US adults had used acupuncture in the previous year [7].

The present study is designed to evaluate the effect of 8 weeks of treatment and 4 weeks of follow-up of true acupuncture compared with sham acupuncture as a treatment adjunct in OA.

## 2. Methods

### 2.1. Study Design and Subjects

We conducted a randomized, controlled trial with blinded evaluation and statistical analysis of the results. It was carried out from July 2002 to October 2003 at the Department of Orthopaedics “B” of the Tel Aviv Sourasky Medical Center, a large university-affiliated institution.

The Complementary Medicine Unit applied the following criteria for inclusion in the study: (i) suitable candidates had to be aged 45 years or older; (ii) were diagnosed as having OA of the knee of at least 6 months duration; (iii) had been suffering from moderate to severe pain during most days throughout the past month for which they had used analgesics for at least 1 month (iv) and were willing and able to complete the study protocol. The exclusion criteria were intra-articular corticosteroid injection into the knees within 4 weeks preceding the study and severe unstable chronic illness (e.g., congestive heart failure, chronic renal failure, cancer).

The patients were divided into either an intervention group that was treated with acupuncture plus standard therapy (e.g., NSAIDs) or a control group that was given sham acupuncture plus standard therapy. Treatment assignment was randomized by a simple randomized allocation method at the beginning of the study. Fifty-five pieces of paper were prepared—half of them containing the word “acupuncture” and half the word “no acupuncture”. The papers were randomly selected and numbered consecutively in a random table held by the research coordinator. Only the acupuncture therapist applying the treatment was informed by the research coordinator of group assignment and he did not participate in any phase of the subsequent evaluation. We took precautions to maintain the confidentiality of the data of the participating patients.

The Hospital Committee for Research Ethics approved the study. All enrolled patients gave their informed consent to participate.

### 2.2. Treatment

A team of accredited acupuncture practitioners selected acupuncture points on the basis of Traditional Chinese Medicine (TCM) treatment methods found to be effective for osteoarthritis of the knee. The protocol points were chosen upon TCM diagnosis in order to recognize the meridians involved in each individual patient. Upon diagnosis the involved meridians were chosen:

1. All patients were needled on the following Acupuncture points: GB34 on opposite side, SP5, Heading, ST35, Xi Yan on the painful side and LI11 or close Ah-shi point on the opposite side.
2. Shu Stream point, which is classically indicated for pain of the joints, was needled on the meridian involved with the knee pain (i.e., when the stomach meridian was involved; The chosen point was ST43, when the kidney meridian the chosen point was KI3).
3. A local point around the knee was added according to the treated meridian (i.e., ST34 to treat pain on the stomach meridian, KI10 to treat pain on the kidney meridian).

The standard acupuncture intervention entailed the insertion of exposable sterile 0.16-mm thick needles manufactured by “Seirin” Co., Japan, and imported by “Medicin Bom” Co., with Israeli Health Department approval. Acupuncture was performed after alcohol wipe of the skin at the specific points. Needles were left in place for a period of 20 min and manually manipulated every 5 min.

The same team carried out the sham acupuncture at the same frequency and according to the same protocol as that used for the intervention group but without insertion of needles into the skin. An empty needle tube was taped to the skin at acupoints to produce sensations similar to those of needle insertion, after which the needles were inserted into a piece of adhesive foam taped to the skin. The sham procedure was similar to the method of Lao et al. [8].

Both real and sham treatments were carried out twice weekly for 8 weeks. Evaluations (detailed below) were carried out at weeks 0, 8 and 12.

### 2.3. Blinding Procedure

**Figure 1:**
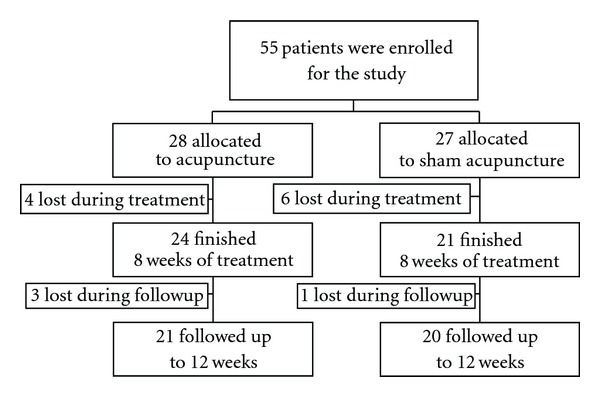
Flow chart of the distribution of the study cohort.

We used a second questionnaire to test the patients' awareness of having been allocated to either real or sham acupuncture groups in order to confirm the validity of the blinding procedure. All participants were asked to report which treatment they believed they had received at the end of the 8-week study period (Figure 1).

### 2.4. Measurements

Our primary outcomes measures were changes in the “Knee Society Score (KSS)” [9] for knee, function and pain self-ratings, on a 10-point Likert scale, at therapy initiation, at 8 weeks and at 12 weeks (1 month after end of treatment). Patient satisfaction and validity of sham acupuncture were assessed as secondary endpoints.

### 2.5. Statistical Analysis

Comparison of demographic and clinical variables between the intervention and sham groups was performed by using the Chi-square and *t*-tests. Treatment satisfaction was compared between groups using the non-parametric Mann-Whitney test. Repeated measures one-way analysis of variance (ANOVA) using the mixed model was carried out to assess group differences and changes over time. Intention to treat procedure was used. Analysis of variance was performed using repeated measures analysis of variance, assuming that missing data are missing at random (MAR) in order to include incomplete or missing patients' data.

Comparisons between patients who completed treatment and dropouts were performed by using Chi-square and *t*-tests. Significance for all tests was set at *P* < .05. All the statistical analyses were performed using SAS for Windows version 9.13.

## 3. Results

Recruitment of patients (*n* = 55) took place between July 2002 and October 2003.  Table 1 lists the baseline characteristics of the patients: there were no clinically relevant differences in the variables that were analyzed at baseline.

Patients (4 of 10) who did not complete the treatment protocol were in the intervention group: one left due to pain; two due to surgery and one for personal reasons. Six dropouts were in the control group: four left because of pain; and two for personal reasons. The only difference between the groups was their KSS function score which was lower in the acupuncture group but without statistical significance (*P* = .06). Four more patients were lost during the follow-up.

The ANOVA showed a significant improvement in knee scores, functional scores and pain scores in all patients after 16 treatments compared to baseline (*P* < .05). After 8 weeks (closure of study) of interventional or sham therapy, there were no significant differences between the two groups in the KSS knee score (*P* = .15), KSS pain score (*P* = .70) or KSS function score (*P* = .23). Only after 12 weeks (i.e., at the 1-month follow-up after treatment) did significant differences emerge between them in the KSS knee score (*P* = .036) and the KSS function score (*P* = .01). No post-treatment adverse effects were reported (Table 2).

Patient satisfaction was measured on a 5-point Likert scale (5 = highly, 1 = not at all). Patient satisfaction in the intervention group (mean score 4.87 ± 0.52) was significantly higher (*P* = .005) than that of the sham group (mean score 3.75 ± 1.12).

Our analysis showed that the blinding procedure was applied successfully. Both the evaluation of the results and the statistical analyses were carried out in a blind fashion.

## 4. Discussion

The results of our study demonstrated that traditional Chinese acupuncture is safe and effective for enhancing physical function in elderly patients with OA of the knee. Unexpectedly, our results demonstrated that significant differences between the intervention and placebo groups in KSS knee scores were not manifest until a minimal period of 12 weeks (8 weeks of therapy and 1 month of follow-up).

Many studies have explored the physiologic processes underlying the clinical effects of acupuncture (reviewed in 10), among them: the release of neurochemicals, such as endogenous opioids [10, 11], segmental nervous system effects (gate theory) [12, 13], autonomic nervous system regulation [14, 15], local effects on brain function [16, 17] and other effects mediated through nervous system [18, 19]. Further studies are required to investigate the precise mechanism underlying the delayed of acupuncture observed in this study.

In a recent systemic review, White et al. [20] showed that acupuncture had a beneficial effect on pain and function in patients with chronic knee pain either in the short-term (2–15 weeks) and long-term (26–52 weeks) treatment. In a large phase III randomized clinical trial, Berman et al. [21] found that the treatment effects of acupuncture in the context of OA were seen only after 12 weeks, resembling the therapeutic effect observed for slow-acting symptomatic drugs for OA, such as glucosamine. Another recent large clinical trial [22] demonstrated the beneficial effect of acupuncture after 8 weeks of treatment. However, with respect to duration of the effect, when evaluated 18 weeks or 44 weeks after end of trial, no advantage was found to the intervention group compared with the sham acupuncture.

In this study, the maximal effect of acupuncture was seen after 12 weeks. Importantly, our results demonstrated that the effect of acupuncture was preserved during the period of 4 weeks following termination of treatment, while KSS knee, function and pain scores improved in the intervention group, they dropped in the sham group. Our study is in concordance other trials in which acupuncture was found to have a long lasting effect that was seen 3 and 6 month after end of treatment [23, 24].

We have used in our study manual acupuncture. Several studies had compared efficacy of manual acupuncture versus electro-acupuncture [25–27]. In the treatment of chronic low back pain auricular, electro-acupuncture was found to relieve pain more effectively than conventional manual auricular acupuncture [26]. Napadow et al. [27] demonstrated with fMRI that both techniques involved effect on limbic system although differences was found in underlying neurobiological mechanisms. In the context of osteoarthritis both large phase III studies using either electro-acupuncture manipulation [21] or manual manipulation showed [22] efficacy of acupuncture. Additional studies are required which protocol is more beneficial for OA of the knee.

We have used the KSS scores, which are widely accepted objective measuring tool to assess knee status in patients waiting for a knee arthroplasty [28–31]. Lingard et al. compared validity and Responsiveness of the Knee Society Clinical Rating System in Comparison with the SF-36 and WOMAC, used in recent studies [22, 23], and concluded that KSS pain and function scores had moderate to strong correlations with the corresponding pain and function domains of the WOMAC and SF-36 (*r* = 0.31–0.72) [32]. The KSS scores assess mechanical parameters which are related to the knees (both functional and clinical components), such as ROM and stability, which are not addressed in the WOMAC score that records mainly the more subjective, patient-relevant outcomes such as pain, stiffness and difficulty of function.

### 4.1. Limitations of the Study

The number of patients enrolled in this study is relatively small and there were no controls of possible confounders associated with the behavior of the therapist, such as the amount of time spent with each patient and effect of therapist-patient communication.

In conclusion, acupuncture treatment is an effective and safe adjunctive therapy to conventional care for elderly patients with OA of the knee. A minimal period of 12 weeks which included 1 month of follow-up was required to achieve significant differences between the intervention and sham groups in KSS knee scores. Future research should determine the optimal duration of acupuncture treatment in the context of OA.

## Figures and Tables

**Table 1:** Baseline comparison of the randomized groups by treatment type and completers versus dropouts (*n* = 55).

Baseline data	Total randomized groups		Completers versus dropouts	
	Intervention group (*n* = 28)	Control group (*n* = 27)	Completers (*n* = 41)	Drop outs (*n* = 14)
Mean age, year (SD)	70.3 ± 10.2	72.2 ± 7.2	71.7 ± 8.6	69.5 ± 9.8
Females, *n* (%)	21 (75)	17 (63)	26	12
Males, *n* (%)	7 (25)	10 (37)	15	2
KSS knee score	52.3 ± 18.5	50.5 ± 19.2	50.9 ± 17.8	53.2 ± 21.9
KSS pain score	16.3 ± 12.1	17.3 ± 10.0	16.9 ± 11.3	16.1 ± 10.4
KSS function score	61.1 ± 20.2	48.7 ± 19.9	58.0 ± 19.4	44.2 ± 26.1

**Table 2:** Bivariant analysis by intention to treat.

	8 weeks		12 weeks (1-month follow-up)	
	Mean (SD)	*P*-value	Mean (SD)	*P*-value
KSS knee score				
Acupuncture	61.6 (16.3)	.15	63.54 (17.4)	.036
Sham	56.8 (17.5)		53.6 (17.1)	
KSS pain score				
Acupuncture	23.7 (10.6)	.7	24.0 (13.2)	.31
Sham	24.4 (11.4)		21.1 (12.7)	
KSS function score				
Acupuncture	65 (17.5)	.23	67.4 (24.2)	.01
Sham	59.7 (20.3)		54.7 (15.0)	

Data are mean (SD) scores.

*KSS*, Knee Society Knee.
